# High Rates of Mental Health Disorders in Civilian Employees Working in Police Organizations

**DOI:** 10.3389/fpsyg.2020.01031

**Published:** 2020-05-28

**Authors:** Liana Lentz, Peter H. Silverstone, Yasmeen I. Krameddine

**Affiliations:** Department of Psychiatry, Faculty of Medicine and Dentistry, University of Alberta, Edmonton, AB, Canada

**Keywords:** civilian, police officer, law enforcement, organization, mental health

## Abstract

Working in a police organization often involves being exposed to potentially traumatic events and stressful circumstances regardless of occupation or rank. Police mental health is a public health concern, but the mental health of civilian employees working in police organizations has been much less studied. The current study aims to compare the frequency of mental health conditions in both police officers and civilians. This was evaluated by measuring mean scores on several mental health screening tools including scales to determine symptom severity for posttraumatic stress disorder (PTSD) with the PTSD Checklist – PCL-5, depression with the Patient Health Questionnaire-9 (PHQ), general anxiety with the Generalized Anxiety Disorder 7-item scale (GAD-7), and alcohol use with the Alcohol Use Disorders Identification Test (AUDIT). The total potential population was 1,225 civilian employees and 3,714 police officers, of which 513 (10%) participated. Of these, 201 (16%) were civilians, and 312 (8%) were police officers (p<0.001). In the study population, 26% screened positive for any mental health disorder. Somewhat surprisingly, we found significantly more civilians (32.8%) than police officers (22.7%) met diagnostic criteria. We also found that civilian participants had higher mean scores in measures of PTSD, anxiety, and depression, although only for depression did this reach statistical significance. Civilians were 1.7 times more likely to screen positive for depression compared to police officers, a statistically significant difference. In contrast, police officers demonstrated statistically higher scores for alcohol use than civilians. One limitation of this study is that the data reflects responses from only a minority of the overall population and, therefore, may not accurately reflect the frequency of mental health issues in the total police organization including civilian employees. Nonetheless, the results strongly suggest that the mental health of all employees can be negatively impacted by working in a police environment, and this is important given the growing number of civilians employed within police organizations. These findings support initiatives aimed at destigmatizing mental health disorders, improving stress management, and increasing access to mental health care on an organization-wide basis, and not just limited to front-line police officers.

## Introduction

Working in a police organization increases the risk of exposure to potentially traumatic events or disturbing content, regardless of occupation or rank. It is well documented that police officers are exposed to traumatic situations frequently in the course of their duties, which can lead to occupational stress injuries such as depression, anxiety, PTSD and substance abuse (Carleton et al., 2018). The effects of trauma exposure, whether acute or cumulative, direct or vicarious, can not only affect the individual but also their families (Ricciardelli et al., 2018) and their organization through increased sick time and increased injury risk (Allodi and Montgomery, 1979; Bedno et al., 2014). Much of the existing research, however, focuses on disaster or critical incidents rather than the accumulation of daily exposures to negativity, hardship, interpersonal violence, and injury (McFarlane et al., 2009; Berger et al., 2012). Less studied is the mental health of civilian employees in police organizations who are secondarily exposed to often gruesome or disturbing content. Their jobs within law enforcement, even as civilians, can be demanding and require employees to make emotional connections with citizens or suppress emotions when being exposed to information about crime (McCarty and Skogan, 2012).

Civilian personnel working within a police organization include those who perform roles such as clerks, communications staff, managers, and other professionals who perform duties that aid the core function of the police service (Conor et al., 2019). Civilians conduct police work that does not require the authority, specialized training, or credibility of a sworn police officer, including investigative assistance, security, and working in holding cells (Taylor et al., 2006; Kiedrowski et al., 2017). In Canada, civilians and police officers are equally present in detention, cell block security, and prisoner escort services (Kiedrowski et al., 2017). While many civilians do not directly deal with the public, those working in public positions may encounter individuals charged with an offense, putting them at risk for physical as well as traumatic psychological injury. A strategic analyst, for example, is responsible for collecting, organizing, collating, analyzing, and developing information from various investigative, operational, and intelligence sources to assist the police organization in meeting their strategic, tactical, and operational objectives (Taylor et al., 2006). Additionally, call takers and dispatchers are exposed to traumatic calls; working in high-pressure environments with little downtime, inadequate debriefing after stressful calls; inappropriate training for mental-health-related calls; and being exposed to verbally aggressive callers (Smith et al., 2019).

The proportion of civilians in police organizations has been consistently increasing (Kiedrowski et al., 2017; Conor et al., 2019). Since 2003, in Canada, the United States (US) and Great Britain, the number of civilian employees has grown twice as quickly as that of sworn officers (42% vs 21%). In Canada in 2018, the number of full-time equivalent personnel in civilian roles grew by 1,998 or 7% over the previous year and has been steadily on the rise since data collection began in 1962. Among such employees, women occupy 57% of civilian positions overall (Kiedrowski et al., 2017), and in North America, women account for 71% of civilian personnel within both Canadian (Conor et al., 2019) and American (McCarty and Skogan, 2012) police services. Furthermore, as the number of civilian employees continues to grow, it is evident not only that civilians are an essential component of a police service but that there are also a growing number of civilian workers that are exposed to activities that may put them at increased risk of occupational or organizational stress injuries similar to those incurred by police officers.

Though civilian employees and police officers cannot be directly compared, it is important to compare the two cohorts in the same organization for differences in mental health. This may inform policy on how to work with these two distinct groups in a single organization. The purpose of this study was to compare the mean scores on selected mental health screening tools between police officers and civilian employees at the same location. Also examined was the proportion of police officers and civilians who screen positive for several mental health disorders, including posttraumatic stress disorder (PTSD), depression, general anxiety disorder, and alcohol use. Additionally, the rates of positive screens in civilian employees was compared with those of the general population.

## Materials and Methods

This was a cross-sectional study of all employees working in two Royal Canadian Mounted Police (RCMP) divisions in Western Canada, representing all police officers and civilian employees within the provinces of Alberta and the Northwest Territories (NWT). The population was composed of 1,225 (25%) civilian employees and 3,714 police officers. An email was sent to all employees inviting them to complete a web-based self-report survey. The survey was in English and included several validated psychological tools used to screen for mental disorder symptoms. The data was obtained in 2019 from April 12 to June 15 (Alberta) and August 1 to September 30 (NWT). Participation in the survey was voluntary and anonymous. Those who elected to participate created a unique personal identifier to remain anonymous and unidentifiable to the researchers. Ethical approval for this study was obtained from the University of Alberta Health Research Ethics Board, ethics review number Pro00073277.

### Subjects and Data

A total of 201 (16%) civilians and 312 (8%) police officers responded to the survey. Thus overall, 10% of potential participants responded. A significantly higher proportion of civilians responded to the survey compared to police officers (*p* < 0.001). Of those who took part, 73% completed the entire survey, with the remaining 27% completing only part of the survey. Data from anyone who started the survey was included. The study sample consisted of 250 females and 263 males, where 68% of the civilian sample were females, and 32% of the police officers were females.

### Screening Tools

Several screening tools were included in the survey to determine scores on scales of PTSD, anxiety, depression, and alcohol use. The scales used were the PTSD Checklist (PCL-5) (Blevins et al., 2015; Ashbaugh et al., 2016), Generalized Anxiety Disorder 7-item scale (GAD-7) (Robert et al., 2006; Beard and Björgvinsson, 2014), Patient Health Questionnaire-9 (PHQ) (Hyphantis et al., 2015; Beard et al., 2016; Christensen et al., 2017), and Alcohol Use Disorders Identification Test (AUDIT) (Saunders et al., 1993). Additionally, individual resilience was measured using the Connor-Davidson Resilience Scale (CD-RISC) (Connor and Davidson, 2003). A descriptive analysis was conducted that included mean scores on each screening tool as well as frequencies and proportions of positive screens. Positive screens were indicated by previously established cut-off scores: the PCL-5 required a score of >32 to indicate likely PTSD; the GAD required a score of >9 (Swinson, 2006) to indicate likely anxiety disorder; the PHQ-9 required a score of >9 to indicate likely depression (Manea et al., 2015), and the AUDIT required a score of >15 to indicate likely alcohol abuse disorder (Gache et al., 2005).

### Statistical Analysis

Participants were grouped according to police or civilian status as well as demographic characteristics, including sex, age, marital status, and the highest level of education. Cross-tabulations were used to determine the frequency of positive screens for each mental health disorder. Proportions between groups were statistically compared using the Fisher’s exact statistic and odds ratios with 95% confidence intervals. Continuous variables were compared using a *t*-test to obtain the significant differences between the means. Logistic regression models were conducted to assess associations between demographic covariates and screening positive for each mental disorder. The data analysis was conducted using SAS software, Version 9.4 (SAS Institute Inc, Cary, NC, United States).

## Results

Overall, 26% of the sample screened positive for any mental health condition. Compared to police officers (22.7%), significantly more civilians (32.8%) scored positive for any mental health condition (*p* = 0.01). This is much higher than the general Canadian population, where 10.1% reported symptoms consistent with at least one mental health or substance use disorder (Pearson et al., 2013). Since those in the workforce are generally healthier and have lower mortality than the general population (McMichael et al., 1974), it would be expected that the civilians in this study demonstrate a lower prevalence of mental health disorders when compared to the general public. This demonstrates that the mental health of civilians working in a police organization is a public health concern. A univariable analysis, adjusted for all other demographic factors, indicated that civilians were 1.67 (95% CI 1.12 to 2.47) times more likely to screen positive for any mental health disorder compared to police officers. A comparison of sociodemographic characteristics and positive screens for any mental health disorder is summarized in Table 1.

**TABLE 1:** Association between any positive screen and sociodemographic variables.

	Civilian		Police	
	Any positive screen^1^ *n* (%)	Odds ratio 95% CI	Any positive screen *n* (%)	Odds ratio 95% CI
Sex				
Male	11 (35.5)	1	52 (22.4)	1
Female	55 (32.4)	0.87 (0.39 to 1.94)	18 (22.5)	1.01 (0.55 to 1.85)
Age Group				
20–29	12 (40.0)	1	4 (26.7)	1
30–39	17 (29.3)	0.62 (0.25 to 1.57)	24 (19.7)	0.67 (0.20 to 2.30)
40–49	22 (38.6)	0.94 (0.38 to 2.33)	33 (26.6)	1.0 (0.30 to 3.35)
50–59	13 (27.7)	0.57 (0.22 to 1.51)	10 (21.7)	0.76 (0.20 to 2.92)
60–69	2 (18.2)	0.33 (0.06 to 1.82)	0 (0.0)	0
Marital Status				
Single	10 (31.3)	1	7 (22.6)	1
Married	34 (31.5)	1.01 (0.43 to 2.37)	52 (22.7)	1.01 (0.41 to 2.47)
Common Law	8 (25.8)	0.77 (0.26 to 2.30)	8 (22.9)	1.02 (0.32 to 3.22)
Divorced/Widowed	13 (46.4)	1.91 (0.67 to 5.47)	3 (20.0)	0.86 (0.19 to 3.92)
Education				
High School	18 (45.0)	1	12 (17.9)	1
College	17 (23.3)	0.37 (0.16 to 0.8	29 (29.3)	1.90 (0.89 to 4.06)
Undergraduate	27 (39.1)	0.79 (0.36 to 1.73)	30 (22.7)	1.35 (0.64 to 2.84)
Graduate Degree	3 (23.1)	0.33 (0.08 to 1.38)	0 (0)	0
Work Location				
Urban	19 (32.2)	1	29 (15.8)	1
Rural	48 (33.8)	0.93 (0.49 to 1.78)	42 (32.6)	0.98 (0.57 to 1.68)

*^1^Any positive screens include respondents who screened positive on any of the established mental disorder (i.e., posttraumatic stress disorder – PTSD), major depressive disorder, generalized anxiety disorder, alcohol abuse) screening tools.*

Mean scores for screening tools are summarized in Table 2. Civilians scored significantly higher on the PHQ-9 (*p* < 0.01) compared to police officers, while police officers scored significantly higher on the AUDIT scale (*p* < 0.01). No statistically significant differences in mean scores were found for PCL-5 or GAD-7; however, the mean scores for civilians were higher for both compared to police officers. There was no significant difference in mean scores on the CD-RISC, indicating no measured differences in resiliency scores.

**TABLE 2:** Screening tool mean scores.

Screening tool	Civilian mean (SD)	Police mean (SD)	*p*-value
PCL 5	19.7 (16.9)	18.7 (17.2)	0.57
GAD	6.6 (5.5)	5.9 (5.2)	0.16
AUDIT	3.5 (3.6)	4.6 (4.7)	0.01*
PHQ	8.4 (6.0)	7.0 (5.6)	0.01*
CD-RISC	70.0 (14.4)	71.0 (16.0)	0.53

*^∗^ denotes that the mean civilian score was statistically different than the mean police score.*

Sex was not a significant indicator of a positive screen for any of the four screening tools (Table 3). Civilians were significantly more likely to screen positive for depression (OR, 1.67; 95% CI, 1.12–2.47) compared to police officers (Table 4).

**TABLE 3:** Positive mental health screening stratified by sex.

Screening tool	Civilian *n* (%)		*p*-value	Police *n* (%)		*p*-value
	Male	Female		Male	Female	
PCL 5	9 (9.7)	32 (34.4)	0.23	41 (44.1)	11 (11.8)	0.49
GAD	11 (10.4)	39 (36.8)	0.17	42 (39.6)	14 (13.2)	1.00
AUDIT	18 (4.8)	119 (31.7)	0.40	191 (45.5)	69 (16.4)	0.31
PHQ	18 (5.4)	100 (29.9)	0.83	163 (48.8)	53 (15.9)	1.00

**TABLE 4:** Unadjusted odds ratios for positive screens for civilians compared to police officers.

Screening tool	Odds ratio	95% Confidence interval	*p*-value
PCL-5	1.23	0.79–1.94	0.36
GAD-7	1.49	0.97–2.29	0.07
AUDIT	0.37	0.10–1.34	0.13
PHQ	1.67	1.12–2.47	0.01*

Proportionally, civilians demonstrated consistently higher numbers of positive screens for depression, anxiety, and PTSD, with the proportion of civilians screening positive for anxiety and depression reaching statistical significance. There were no significant relationships between demographic factors and positive mental health screens. The single best predictor of an increased likelihood of screening positive for any mental health disorder was being a civilian employee in the police organization.

## Discussion

This study aimed to compare the mental health of police officers and civilian employees working in the same organization. More specifically, this study used mean scores on four mental health screening tools to determine the frequency that police officers and civilians screen positive for PTSD, depression, anxiety disorder, and excessive alcohol use.

In this study, 26% of the sample screened positive for a mental health disorder. Civilian participants demonstrated mean scores that were consistently higher than those demonstrated by police officers for PTSD, anxiety, and depression, whereas police officers demonstrated a higher mean score for alcohol use disorder. Mean scores for depression were significantly higher for civilians, whereas police officers had a significantly greater mean score for alcohol use disorder. The only statistically significant difference in risk was for depression, where civilians were 1.7 times more likely to screen positive for depression compared to police officers.

Relatively little research has investigated the mental health status of civilians working in a police organization (McCarty and Skogan, 2012). Carleton et al. compared several public safety personnel (PSP) groups according to mental health outcome screening. Apart from the PCL-5, the mean scores found in that study are not remarkably different from those found here, they differ in that police officers demonstrated increased mean scores on the PCL-5, GAD-7, and PHQ while civilians scored slightly higher on AUDIT. This is an interesting comparison since our results found the reverse, and both studies surveyed RCMP employees. The differences may lie in that the current study included all civilian employees, whereas Carleton et al. only examined one specific civilian group; call center operators and dispatchers. Additionally, our sample was geographically limited to Alberta and the NWT, whereas Carleton used a national sample. These differences in results are also mirrored when comparing the proportions of positive screens between police officers and civilians. However, when compared to all the PSP groups surveyed, Carleton’s results are similar to those in this study in that civilian employees, compared to police, reported slightly higher mean scores and slightly more frequent positive screens for most mental disorders except for AUDIT, which was slightly lower.

The differences between police officers and civilian employees may also reflect what is commonly referred to as “burnout”, and it can affect a worker’s motivation and health (McCarty and Skogan, 2012). Burnout is a state of exhaustion in response to chronic job stressors, which can be related to job tasks, interpersonal relationships, or emotions (Maslach et al., 2001; World Health Organization, 2018). Burnout and stress can lead to illness, mood changes, alcohol use, and sleep disturbances in the short-term and perhaps even to cardiovascular disease and psychological disorders in the long-term (McCarty et al., 2011). Rates of burnout and job dissatisfaction amongst civilian personnel in police organizations are high (Kiedrowski et al., 2017). Since burnout is a multidimensional condition consisting of emotional exhaustion, depersonalization, and a reduced sense of accomplishment (Maslach and Jackson, 1981), the feelings of helplessness, horror, or fear in reaction to a call experienced by 1/3 of police dispatchers may contribute to their decreased mental health and burnout (Pierce and Lilly, 2012). This could be one of the causes of the increased rates of mental health problems we identified, although differences in the degree of burnout between civilian and police are uncertain and may not be very large (McCarty and Skogan, 2012).

Research has also suggested several organizational features can influence an employee’s mental health, such as supervisor relations, group morale and cohesion, administrative procedures, workload, shift work, availability of resources, and interpersonal conflict (McFarlane and Bryant, 2007). Attention is required to enhance the occupational and organizational characteristics of police services, and improve the police culture and organizational climate (Randall and Buys, 2013). It is suggested that civilian job satisfaction is related to management and organizational factors more than it is to the individual employee and that the rate of civilian staff suffering from physical or psychological injuries is equal to or exceeds the rate of sworn officers (Kiedrowski et al., 2017) which are consistent with our findings.

More specifically, working in a bureaucratic structure of law enforcement may not only be foreign to a civilian but stressful as well. Since the rank structure of a police service makes it more of a “paramilitary” organization, civilians may feel that they do not belong or may be treated as “the other” or lesser than when compared to police officers (Chess, 1960; McCarty and Skogan, 2012; Ratcliffe, 2005). Feeling like one belongs is a fundamental human need (Baumeister and Leary, 1995; Gere and MacDonald, 2010). It has been found that a sense of belonging to an occupational group and the perception of social support can decrease feelings of burnout (McCarty and Skogan, 2012) and increase resilience (McAllister and McKinnon, 2009; Howe et al., 2012). In contrast, a low sense of belonging is strongly associated with higher levels of depression (Hagerty et al., 1996; Hagerty and Williams, 1999). Thus, organizational issues may be an essential factor in the high rates of mental health concerns in civilian employees.

This study had limitations. The first is the relatively small response rates within both groups, 8% of police officers and 16% of civilians. While such low response rates are typical of extensive questionnaires, it is possible that participants are not representative of the wider population. A second limitation of this study is that we cannot discern how much the work tasks or environment influenced the outcome measure scores, or how much these were influenced by conditions that pre-existed employment. Thirdly, we were not able to determine if non-work issues or other personal circumstances may have influenced an individual’s responses. Lastly, participants who chose to complete the questionnaire may have self-selected themselves if they felt that they were struggling with mental health issues. Nonetheless, given that the frequency of positive screening for mental health disorders is much higher than that of the general population, it is appropriate to conclude that the police organizational workplace may have played a significant role in the mental health status of participants.

## Conclusion

The results of this study indicate that both police officers and civilian employees in a police organization have higher rates of mental health disorders compared to the general population, with civilian employees having the highest rates. This has several practical implications: firstly, police officer mental health is a current recognized concern and should be extended to include civilian employees. It is, therefore, important that police organizations consider the health of all employees, and initiatives aimed at destigmatizing mental health disorders, improving stress management, and increasing access to mental health care should not be limited to police officers but should be organization wide. Peer support or mentoring programs should also be available to civilian employees. It must be the responsibility of police organizations to encourage inclusivity of all employees in order to increase a sense of belonging and cohesion within the workplace and to reduce mental health risks for civilian employees.

The results also demonstrate the need for specific training and organizational support to minimize risks to the mental health of all police organization employees. Such support should be based upon current evidence, and its effectiveness measured to confirm that it improves mental wellness for both police officers and civilian employees of law enforcement organizations and should involve both short-term and longer-term outcomes.

## Data Availability Statement

The datasets generated for this study are available on request to the corresponding author.

## Ethics Statement

Ethical approval for this study was obtained from the University of Alberta Health Research Ethics Board, ethics review number Pro00073277. The patients/participants provided their informed consent to participate in this study.

## Author Contributions

PS and YK conceived, designed, and were awarded the grant for the concept of the original research. LL and YK advanced the design and delivered the questionnaire. YK collected the data and supervised this project. LL analyzed and interpreted the data and drafted the manuscript. All authors reviewed the manuscript, offered revisions, and gave final approval for submission to publication. All authors agreed to be accountable for all aspects of the work in ensuring that questions related to the accuracy or integrity of any part of the work are appropriately investigated and resolved.

## Conflict of Interest

The authors declare that the research was conducted in the absence of any commercial or financial relationships that could be construed as a potential conflict of interest.
